# Synergistic effect of electromagnetic fields and nanomagnetic particles on osteogenesis through calcium channels and p‐ERK signaling

**DOI:** 10.1002/jor.24905

**Published:** 2021-01-13

**Authors:** Yu‐Mi Kim, Han‐Moi Lim, Eun‐Chul Lee, Ga‐Eun Ki, Young‐Kwon Seo

**Affiliations:** ^1^ Department of Medical Biotechnology (BK21 Plus Team) Dongguk University Goyang‐si Korea

**Keywords:** calcium channel, calvarial defect model, electromagnetic fields, nanomagnetic particle, osteogenesis

## Abstract

Electromagnetic fields (EMFs) are widely used in a number of cell therapies and bone disorder treatments, and nanomagnetic particles (NMPs) also promote cell activity. In this study, we investigated the synergistic effects of EMFs and NMPs on the osteogenesis of the human Saos‐2 osteoblast cell line and in a rat calvarial defect model. The Saos‐2 cells and critical‐size calvarial defects of the rats were exposed to EMF (1 mT, 45 Hz, 8 h/day) with or without Fe_3_O_4_ NMPs. Biocompatibility was evaluated with MTT (3‐(4,5‐dimethylthiazol‐2‐yl)‐2,5‐diphenyltetrazolium bromide) and LDH (lactate dehydrogenase) assays. This analysis showed that NMP and EMF did not induce cell toxicity. Quantitative reverse‐transcription polymerase chain reaction indicated that the osteogenesis‐related markers were highly expressed in the NMP‐incorporated Saos‐2 cells after exposure to EMF. Also, the expression of gene‐encoding proteins involved in calcium channels was activated and the calcium concentration of the NMP‐incorporated + EMF‐exposed group was increased compared with the control group. In particular, in the NMP‐incorporated + EMF‐exposed group, all osteogenic proteins were more abundantly expressed than in the control group. This indicated that the NMP incorporation + EMF exposure induced a signaling pathway through activation of p‐ERK and calcium channels. Also, in vivo evaluation revealed that rat calvarial defects treated with EMFs and NMPs had good regeneration results with new bone formation and increased mineral density after 6 weeks. Altogether, these results suggest that NMP treatment or EMF exposure of Saos‐2 cells can increase osteogenic activity and NMP incorporation following EMF exposure which is synergistically efficient for osteogenesis.

## 1 INTRODUCTION

Electromagnetic fields (EMFs) have been widely used in the stimulation of wound healing and for relieving pain. Numerous attempts have been made to evaluate the effects of EMFs on cellular activity and proliferation.^1^, ^2^ Especially, electromagnetic stimulation has been studied for applications in treating bone disorders and regenerating bone. Several studies have reported that EMFs increase the proliferation of human osteoblasts and osteosarcoma cell lines in vitro.^3^, ^4^, ^5^ Also, it has been reported that exposure to various EMFs (7.5–75 Hz) plays a modulatory role in the osteogenic differentiation of human bone marrow‐derived mesenchymal stem cells with an increase in alkaline phosphatase and osteogenesis‐related genes.^6^, ^7^, ^8^ EMFs have been reported to stimulate healing in disconnected fractures of the tibia, induce bone repair,^9^ and to reduce the healing time following fresh fractures.^10^ This knowledge is based on the discovery of the electromechanical properties of bone,^11^, ^12^ which raised the possibility that electric energy may stimulate bone formation and modify the behavior of bone cells.^13^, ^14^

Also, nanoparticles (NPs) have been widely used in biomedical applications such as drug delivery, biological labels, and the detection of proteins, and many studies have shown that NPs promote the migration and differentiation of cells, thereby inducing stem cell differentiation and stimulating wound healing.^15^, ^16^, ^17^ Kim et al.^18^ showed that Fe_3_O_4_ nanomagnetic particles (NMPs) could affect cell–substrate interactions and enhance neurite outgrowth of PC12 cells.^17^ Especially, surface‐modified NMPs are expected to increase their circulation time, aqueous solubility, biocompatibility, and nonspecific cellular uptake and to decrease immunogenicity.^19^ Zhang et al.^20^ reported that PEGylated MNPs not only facilitated cellular uptake into cancer cells but also increased the yield of cell internalization. Additionally, it has been reported that PEGylated NMP increased the wound‐healing effect upon incorporation into human bone marrow‐derived mesenchymal stem cells in injured rat spinal cord.^21^

Recently, synergistic effect studies on cell proliferation and differentiation have been reported using combinations of magnetic NPs and magnetic fields. Researchers manufactured porous hydroxyapatite, poly(ε‐caprolactone) (PCL), and polylactic acid scaffolds containing or coated with iron oxide magnetic NPs (20–40 nm). Then, various cells were inoculated on the scaffold, and exposed to a magnetic field, followed by an evaluation of osteogenesis. The results of these studies showed that osteogenesis efficacy was increased in the scaffolds with a magnetic field relative to the only‐scaffolds groups.^22^, ^23^, ^24^ Also, another study adhered osteoblasts on RGD‐coated magnetic (4.5 µm ferromagnetic) particles and then exposed them to a magnetic field, thus applying direct mechanical stimulation to the cells (*B*_max_ approximately 60 mT). Their Von Kossa staining was strong and their messenger RNA (mRNA) expression levels of osteopontin were high.^25^

However, all of the previous studies evaluated the effect of cell adhesion on NMPs, not the phagocytosis efficacy of NP into the cytosol. We wanted to study the bone regeneration effect after the NMPs were injected directly into the bone defect area, following which the NMPs would be phagocytosed into the surrounding cells, but this method proved to be impossible. Therefore, we manufactured rapidly degradable collagen sponges and inoculated the NMPs just before transplantation because we wanted them to be taken up by the cells.

In the present study, we investigated the synergistic effects of EMF and Fe_3_O_4_ NMP treatment on osteogenic activity. We applied EMFs and NMPs to the Saos‐2 osteoblast cell line and a rat calvarial defect model. The Saos2 cells and the rat calvarial defect model were treated with 50 μg/ml of Fe_3_O_4_ MPs or exposed to a frequency of 45 Hz at an intensity of 1‐mT EMF for 8 h/day and examined whether treatment with Fe_3_O_4_ NMPs in conjunction with exposure to EMFs is more effective in enhancing osteogenic activity.

## 2 MATERIALS AND METHODS

### 2.1 Saos‐2 cell culture

The human osteosarcoma cell line Saos‐2 was cultured with RPMI‐1640 media (Welgene) containing 10% fetal bovine serum (Lonza) and 1% penicillin/streptomycin (Welgene). The cells were cultured at 37°C in a 5% humidified CO_2_ atmosphere in 100‐mm dishes.

### 2.2 EMF exposure

**Figure 1:**
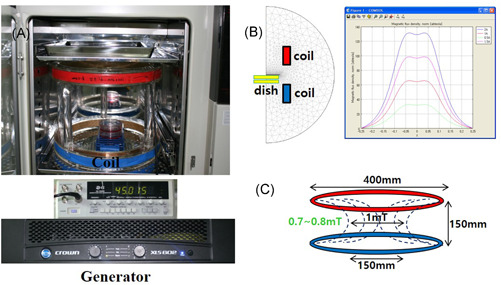
(A) Schematic representation of the sinusoidal electromagnetic field (EMF) device. Simulated magnetic flux density distribution at 45 Hz by COMSOL 3.4. (B) Simulation model and simulated magnetic flux density (I ¼ 200 mA). (C) Schematic diagram of the EMF device and distribution of EMF. The sinusoidal EMF device was placed in an incubator with 5% CO₂ maintained at 37°C [Color figure can be viewed at wileyonlinelibrary.com]

In this study, continuous sinusoidal EMFs (*B*_m_ = 1 mT, *F* = 45 Hz sinusoidal) were used for the experiments (Figure 1). All experimental groups were kept in a cell culture incubator at 37 ± 0.1°C and 5% CO_2_ concentration.

Figure 1B shows the simulated magnetic flux density distribution at 45 Hz by COMSOL 3.4 (magnetic flux density: I ¼ 200 mA), and Figure 1C is a schematic diagram of the EMF device and the distribution of the EMF. The stimulation unit was designed to handle a pair of identical coils measuring 40 cm in diameter assembled in a Helmholtz configuration. The pair of coils operated on alternating current, generating EMF, and the current in the coil was controlled by a function generator (FG‐7020A) and power source (Professional Amplifiers, XLS, Crown Audio Inc.). The cells and experimental rats were applied EMF consisting of 45‐Hz frequency and an intensity of 1 mT for 8 h/day.

### 2.3 Preparation of the Fe_3_O_4_ NMPs

Water‐dispersible and biocompatible Fe_3_O_4_ NMPs were prepared using a method described previously.^20^, ^25^ The monodispersed Fe_3_O_4_ NMPs were dispersed in a nonpolar organic solvent and synthesized using a high‐temperature organic solution phase reaction. Iron(III) acetylacetonate (Fe(acac)_3_, 2 mmol, 99.9%; Sigma‐Aldrich), 1,2‐hexadecanediol (10 mmol, 90%; Sigma‐Aldrich), oleic acid (6 mmol, 99%; Sigma‐Aldrich), oleylamine (6 mmol, 70%; Sigma‐Aldrich), and 1‐octadecene (20 ml, 90%; Sigma‐Aldrich) were mixed and magnetically stirred in a nitrogen atmosphere. The mixture was heated to 200°C for 2 h and then heated to reflux (~300°C) for an additional hour. Ethanol (40 ml) was added to the mixture under ambient conditions, and a black material was precipitated and separated via centrifugation (12,000 rpm, 30 min). The black product was redispersed in hexane in the presence of oleic acid (~0.05 ml) and oleylamine (~0.05 ml). The resulting Fe_3_O_4_ NMPs dispersed in chloroform were encapsulated with a polyethylene glycol (PEG)‐phospholipid shell to make them biocompatible. Typically, 2 ml of the organic dispersible 12‐nm Fe_3_O_4_ NMPs in chloroform (5 mg/ml) was mixed with 1 ml of chloroform solution containing 10 mg 1,2‐distearoyl‐*sn*‐glycero‐3‐phosphoethanolamine‐*N*‐(methoxy(PEG)‐2000; mPEG‐2000 PE; Avanti Polar Lipids Inc.) at a ratio of 5:1. After complete evaporation of the chloroform, the residue was incubated at 80°C in vacuum for 1 h. Then, 5 ml of water was added, which produced a clear and dark‐brown suspension containing the PEG–PE micelles. In this study, 50 µg/ml of Fe_3_O_4_ NMPs were added to the medium.

### 2.4 Characterization of Fe_3_O_4_ NMPs

**Figure 2:**
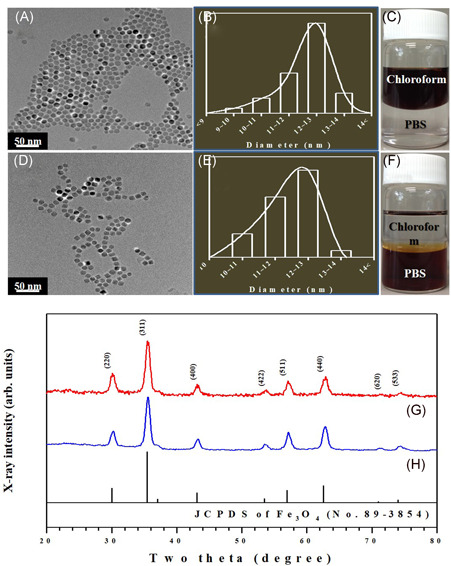
High‐resolution transmission electron microscopy images of (A) the pristine Fe_3_O_4_ nanomagnetic particles (NMPs) deposited from chloroform dispersion on an amorphous carbon‐coated copper grid, and (D) polyethylene glycol (PEG)‐encapsulated Fe_3_O_4_ NMPs deposited from a phosphate‐buffered saline (PBS) dispersion on an amorphous carbon‐coated copper grid. (B,E) The particle size distribution curves and (C,F) dispersions of the pristine Fe_3_O_4_ and PEG‐encapsulated Fe_3_O_4_ NMPs, respectively. X‐ray diffraction patterns of (G) the pristine Fe_3_O_4_ NMPs, and (H) PEG‐encapsulated Fe_3_O_4_ NMPs [Color figure can be viewed at wileyonlinelibrary.com]

The crystal structures of the obtained Fe_3_O_4_ NMPs were studied by powder X‐ray diffraction (XRD) measurements using Ni‐filtered Cu Kα radiation (*λ* = 1.5418 Å, D/MAX 2000 vk/pc; Rigaku) with a graphite‐diffracted beam monochromator. The patterns were recorded at an operating voltage of 40 kV and a current of 20 mA. The morphology and size of the obtained Fe_3_O_4_ NMPs were examined using a JEOL JEM‐3010 high‐resolution transmission electron microscope (HR‐TEM) at an operating voltage of 300 kV (Figure 2A,D).^25^

### 2.5 Proliferation and activity assay of Saos‐2

Cell proliferation was measured with a 3‐(4,5‐dimethylthiazol‐2‐yl)‐2,5‐diphenyltetrazolium bromide (MTT; Sigma‐Aldrich) assay. For the MTT assay, the cells were cultured in a six‐well plate, and each well was supplemented with MTT (3 mg/ml; *n* = 4). The plates were then incubated in the dark at 37°C in an atmosphere containing 5% CO_2_ for 2 h, and the supernatant was aspirated. Dimethyl sulfoxide was added, and the six‐well plate was shaken slowly for 5 min. The absorption was measured at 570 nm.

### 2.6 Lactate dehydrogenase (LDH) assay

LDH activity was measured using an LDH‐LQ kit (Asan Pharmaceutical Inc.). Briefly, after 7 days of culture, 20‐μl culture medium and 50 μl of working solution were mixed and incubated in darkness at room temperature for 30 min. The reaction was terminated by adding 1‐N HCl, and the absorbance was measured at 570 nm.

### 2.7 Reverse‐transcription polymerase chain reaction (RT‐PCR) analysis

The total RNA of the cells was isolated using 500‐μl TRIzol (Sigma‐Aldrich). Subsequently, 100 μl of chloroform was added, and the solution was mixed and incubated for 3 min. After centrifugation (12,000 rpm, 4°C for 15 min), the upper phase was transferred into a new tube, and 500 μl of isopropanol was added. After a 10‐min incubation period and another centrifugation step (14,000 rpm, 4°C for 10 min), the supernatant was discarded. The pellet was washed with 1 ml of 70% ethanol and centrifuged (9500 rpm, 4°C for 5 min). The supernatant was discardedand the pellet was dried. The pellet was dissolved in 20 μl of diethyl pyrocarbonate‐water. Reverse transcriptase reactions were used to synthesize complementary DNA from 1 μg of total RNA using an Advantage RT‐PCR Kit (Clontech). RT‐PCR was routinely performed. The primer sequences used for the RT‐PCR are listed in Table 1, and ImageJ software (National Institutes of Health) was used for the quantitative analysis of RT‐PCR amplicons on the digitized gel images.

**Table 1:** Primers sequences used for reverse‐transcription polymerase chain reaction experiments

Genes	Upstream primer sequence	Downstream primer sequence
GAPDH	5ʹ‐ACCACAGTCCATGCCATCAC‐3ʹ	5ʹ‐TCCACCACCCTGTTGCTGTA‐3ʹ
Collagen1	5ʹ‐GAAAACATCCCAGCCAAGAA‐3ʹ	5ʹ‐CAGGTTGCCAGTCTCCTCAT‐3ʹ
Collagen3	5ʹ‐CAGGTGAACGTGGAGCTG C‐3ʹ	5ʹ‐TGCCACACGTGTTTCCGTGG‐3ʹ
Osteonectin	5ʹ‐CCAGAACCACCACTGCAAAC‐3ʹ	5ʹ‐GGCAGGAAGAGTCGAAGGTC‐3ʹ
Osteocalcin	5ʹ‐AGGGGAAGAGGAAAGAAGGG‐3ʹ	5ʹ‐CCAGGCGCTACCTGTATCAA‐3ʹ
Osteopontin	5ʹ‐TCGCAGACCTGACATCCAGT‐3ʹ	5ʹ‐TCGGAATGCTCATTGCTCTC‐3ʹ
BMP2	5ʹ‐GTCCAGCTGTAAGAGACACC‐3ʹ	5ʹ‐GTACTAGCGACACCCACAAC‐3ʹ
Runx‐2	5ʹ‐CTCACTACCACACCTACCTG‐3ʹ	5ʹ‐TCAATATGGTCGCCAAACAGATTC‐3ʹ
BSP	5ʹ‐CACAGCCTCATCTTCATGG‐3ʹ	5ʹ‐GCATCTCATAGTGCATCTGG‐3ʹ
OPG	5ʹ‐AAAACGGCAACACAGCTCAC‐3ʹ	5ʹ‐AGGGGAAGAGGAAAGAAGGG‐3ʹ
CACNA1G	5ʹ‐CGGCAACTACGTGCTCTTC A‐3ʹ	5ʹ‐GTGACTTCATCTCGTGGGCC‐3ʹ
CACNA1I	5ʹ‐CGTTGTCATAGCGACCCAGTT‐3ʹ	5ʹ‐CACAGCTCTCTTCCCCGAGTGA‐3ʹ

Abbreviations: BMP‐2, bone morphogenic protein‐2; BSP, bone sialoprotein; CACNA1G, alpha 1G subunit of the T‐type voltage‐dependent calcium channel; CACNA1I, alpha 1I subunit of T‐type voltage‐dependent calcium channel; OPG, osteoprotegerin; Runx‐2, Runt‐related transcription factor 2.

### 2.8 Western blot analysis

After 10 days of culture, the cells were collected, washed with phosphate‐buffered saline (PBS) and lysed with using radioimmunoprecipitation assay buffer containing 50‐mM Tris‐HCl, pH 8.0, 150‐mM NaCl, 1% NP‐40, 0.5% sodium deoxycholate, 0.1% SDS (Sigma‐Aldrich), and protease inhibitors (Complete™; Roche Diagnostics) for 10 min at 4°C. The cell lysates were then denatured at 100°C for 5 min. The protein content of the cell lysates was quantified by the bicinchoninic acid assay, and equal amounts of protein per sample were separated by 10% sodium dodecyl sulfate‐polyacrylamide gel electrophoresis and electrotransferred onto a nitrocellulose membrane (Millipore Co.). The membranes were blocked in 5% fat‐free skim milk dissolved in Tris‐buffered saline (TBS) containing 0.1% Tween‐20 (TBS‐T buffer) at room temperature for 1 h. After washing with TBS‐T, the membrane was incubated for 1 h in 10% bovine serum albumin containing the indicated primary antibodies: anti‐BSP, anti‐osteopontin, anti‐osteonectin, anti‐osteocalcin, anti‐versican, anti‐ERK, anti‐p‐ERK, anti‐p38, anti‐p‐p38, and anti‐β‐actin. The blots were incubated with the primary antibodies at a dilution of 1:5000 and then further incubated with horseradish peroxidase‐conjugated secondary antibody for 2 h at room temperature. The membrane was washed in TBS‐T; the blot was visualized with enhanced chemiluminescence reagent (Thermo Fisher Scientific) and photographed using a gel imaging system, ChemiDoc XRS+ (Bio‐Rad). The results were quantified using ImageJ software (National Institutes of Health).

### 2.9 Immunocytochemical analysis

The cells grown on coverslips were fixed using 4% paraformaldehyde for 20 min at 4°C and then washed with 10‐mM Tris‐HCl buffer. Then, they were incubated with the indicated primary antibodies: anti‐osteocalcin (predilution, AM 386; BioGenex), anti‐osteopontin (1:1000 dilution), and anti‐osteonectin (1:500 dilution, AB 1858; Chemicon) for 24 h, followed by development using EnVision Plus Reagent (Dako), diaminobenzidine as a chromogen, and Mayer's hematoxylin as a counterstain. Microscopic images were captured with a Nikon digital camera attached to a Nikon Optiphot‐2 microscope.

### 2.10 Von Kossa staining

The mineralized matrix of the cells was assessed using 5% silver nitrate (Sigma‐Aldrich) under ultraviolet light for 60 min, followed by 3% sodium thiosulphate (Sigma‐Aldrich) for 5 min, and then counterstained with Van Gieson (Sigma‐Aldrich) for 5 min. The mineral was stained black and the osteoid was stained red by this method.

### 2.11 Immunofluorescence

For staining of the intracellular proteins, the cells were fixed and then permeabilized with 0.1% Triton X‐100 (Sigma‐Aldrich) for 5 min on ice. They were incubated with mouse primary antibodies against human osteopontin (1:1000) for 1 h, followed by a fluorescein‐coupled anti‐rabbit IgG secondary antibody for 1 h.

### 2.12 Quantitative colorimetric calcium assay

Colorimetric calcium was measured using a QuantiChrom™ Calcium Assay Kit (Bioassay Systems). Briefly, samples were lysed with Pro‐prep lysis buffer. Following preparation of the samples, they were mixed with working reagent by combining equal volumes of Reagent A and Reagent B. These reagents were equilibrated to room temperature before use. The mixtures were incubated for 3 min at room temperature. The amount of calcium was quantified by absorbance at 570 nm.

### 2.13 In vivo critical‐sized calvarial bone defect model in the Lewis rat

The experiment was approved by the Institutional Animal Care Use Committee (IACUC Approval No. 2019‐023‐1) and performed according to the NIH Guidelines for the Care and Use of Laboratory Animals. Twenty‐five male Lewis rats (10 weeks old) were used for scaffold implantation and EMF exposure in the calvarial defect. The animals were anesthetized with sevoflurane (SevoFlo; Aesica Queenborough Limited) in combination with air and O_2_. A saline‐cooled trephine drill was used to create two defects of 4‐mm diameter in each parietal bone, carefully protecting the dura mater. The rats were randomly allocated into the following groups, and the defects were filled with collagen sponge scaffolds as indicated: (i) sham surgery, (ii) collagen sponge, (iii) collagen sponge soaked in NMP‐incorporated, (iv) collagen sponge + EMF‐exposed, (v) collagen sponge soaked in NMP‐incorporated + EMF‐exposed. The incisions were closed with 4–0 vicryl sutures. The animals were given a subcutaneous analgesic dose of buprenorphine (temgesic, 0.3 mg/kg). The animals were monitored daily for the condition of the surgical wound, animal activity, food intake, and any signs of infection. Animals were euthanized with CO_2_ inhalation at 6 weeks.

### 2.14 Microcomputed tomographic (micro‐CT) analysis

The rats were killed 40 days after surgery, and the specimens were fixed in 10% formalin for 1 week. Micro‐CT scans were taken and analyzed as previously described.^26^ In brief, micro‐CT scans were taken using the Quantum FX micro‐CT X‐ray system (PerkinElmer). Source voltage and current were set at 90 kV and 180 µA, respectively. The exposure time was 316 ms for optimized clearness. An optimized three‐dimensional (3D) cone beam reconstruction algorithm with a cluster volumetric reconstruction (Feldkamp algorithm) was used. A CT‐analyzing program used two‐dimensional (2D)‐cut images to build a 3D model. The scan parameters were 20–100 kV, 10 W with rotation. Full 3D images of the whole calvarial bone were obtained with a cubic size of 34.58 µm. Reconstruction and analyses were performed using NRecon reconstruction® (SkyScanVR) and CTAn 1.8® software (SkyScanVR), respectively. To measure the newly formed bone, the volume of interest was demarcated in each 2D image. Then, microarchitecture parameters, including bone volume (BV), were obtained using CTAn 1.8 software, according to the manufacturer's instructions. To measure bone mineral density (BMD), the attenuation data for the volume of interest was converted into Hounsfield units and expressed as a value of BMD using phantom scans (QRM). BMD values were expressed in terms of grams per cubic centimeter of calcium hydroxyapatite in distilled water.

### 2.15 Statistical analysis

Data were analyzed using one‐way analysis of variance and Student's *t* test. When the *p* value was <.05 or <.01, the difference between means was considered significant (*p < .05, **p < .01). Graphical representations were produced using Sigmaplot 2001.

## 3 RESULTS

### 3.1 HR‐TEM and X‐ray diffraction analysis of NMP

Figure 2A shows an HR‐TEM image of the spherical and uniform pristine Fe_3_O_4_ NMPs with a high degree of crystallinity, in addition to a nonaggregated form. These Fe_3_O_4_ NPs self‐assemble in a hexagonal close‐packed superlattice due to their high degree of uniformity in diameter. The presence of the residual surfactants (oleic acid and oleylamine) on the NP surface keeps them isolated from each other by a coating layer of about 2 nm, which acts as an ideal system for their encapsulation with PEG.

Figure 2D presents an HR‐TEM image of the PEG‐encapsulated Fe_3_O_4_ NMPs that shows no obvious change in core size after PEG encapsulation.^27^ Even though it is hard to identify the PEG encapsulation by HR‐TEM measurements due to it being organic materials, the PEG‐encapsulated Fe_3_O_4_ NMPs were well dispersed in the PBS media without any aggregation.

Figures 2B and 2E demonstrate the particle size distribution (PSD) of the pristine Fe_3_O_4_ NMPs and PEG‐encapsulated Fe_3_O_4_ NMPs. The PSD calculated on about 100 NPs in different images in the bright field mode led to a measured mean diameter of 12.1 ± 0.8 and 12.0 ± 0.8 nm, respectively.

Also, Figures 2C and 2F shows the dispersibility of the pristine Fe_3_O_4_ NMPs and PEG‐encapsulated Fe_3_O_4_ NMPs. The pristine Fe_3_O_4_ NMPs (Figure 2C) can be dispersed in an organic solvent such as chloroform, but after PEG encapsulation (Figure 2F), these NMPs were well dispersed in aqueous media such as PBS for biological applications.

The XRD patterns of the dry powders of the Fe_3_O_4_ NMPs are shown in Figure 2G,H. The position and relative intensity of all diffraction peaks can be indexed to a pure *fcc* phase [space group: *Fd3m* (227)] of Fe_3_O_4_ with cell constants *a* = 8.394 Å, which match well with that of Fe_3_O_4_ (JCPDS No. 89‐3854). After PEG encapsulation occurs on the surface of the Fe_3_O_4_ NMPs, all of the diffraction peaks related to the pristine Fe_3_O_4_ NMPs clearly remain without any distinctive change (Figure 2G,H).^27^ These results support the conclusion that the crystal structure of the Fe_3_O_4_ NMPs does not change after PEG encapsulation.

### 3.2 Morphology of Saos‐2

**Figure 3:**
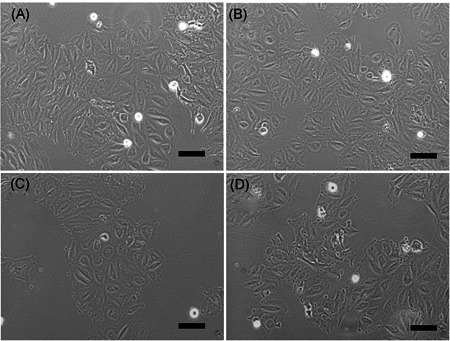
Morphology of the Saos‐2 after electromagnetic field (EMF) treatment. Cultured in maintenance medium (A), magnetic nanoparticle (NMP)‐incorporated (B), exposed to EMF (C), and NMP‐incorporated with exposed EMF (NMP + EMF) (D) for 7 days. Original magnification: (A–D) ×100, scale bar = 100 μm

Figure 3 shows the morphology of the Saos‐2 cells under different culture conditions. The Saos‐2 cells were observed to have a small and polygonal morphology, and morphological changes or necrosis were not observed in any of the experimental groups. Therefore, NMP incorporation, EMF exposure, and NMP incorporation with EMF exposure were not found to induce cytotoxicity.

### 3.3 Effects on proliferation and cytotoxicity assays

**Figure 4:**
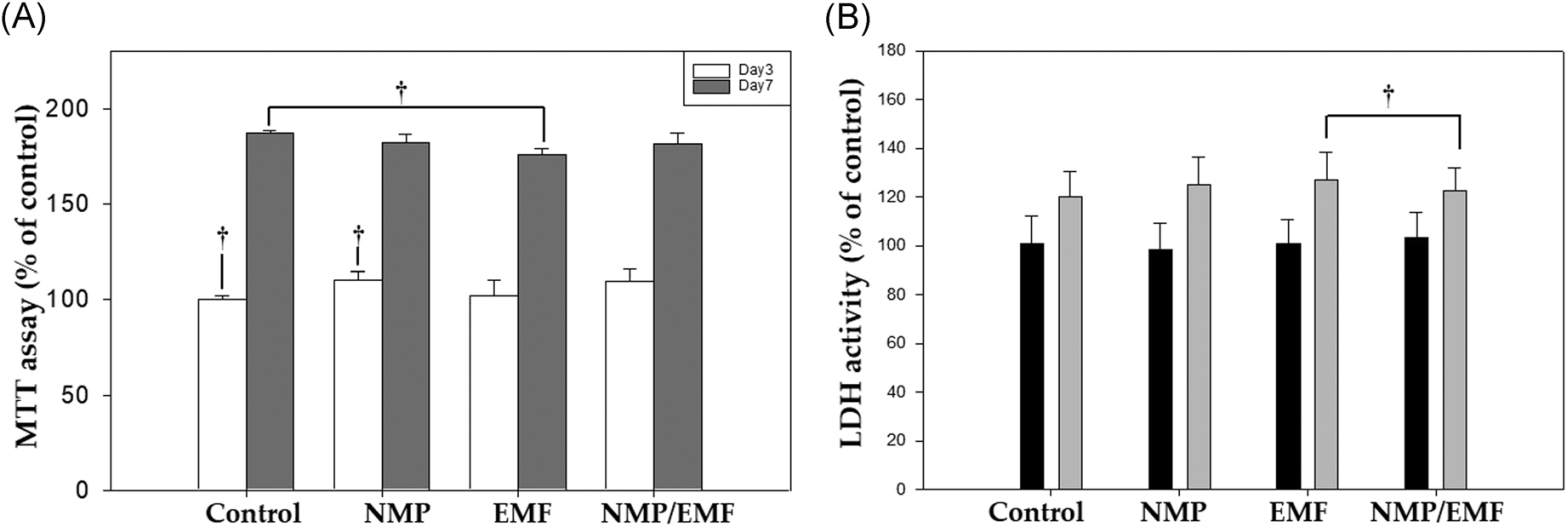
Effect of NMPs and EMF on the proliferation of Saos‐2. (A) MTT assay was performed to evaluate cell viability of Saos‐2 under different conditions (control, NMP, EMF, NMP/EMF) after 7 days. (B) Effect of culture conditions on the cytotoxicity of Saos‐2 after culture for 7 days. Their cytotoxicity was measured by an LDH Assay Kit. EMF, electromagnetic field; LDH, lactate dehydrogenase; MTT, 3‐(4,5‐dimethylthiazol‐2‐yl)‐2,5‐diphenyltetrazolium bromide; NMP, nanomagnetic particle. ^†^ *p* > .05

Biocompatibility of the EMFs and NMPs was evaluated by MTT and LDH assays. The initial seeding cell number in each subculture was the same in each group. Saos‐2 cell proliferation was measured by MTT assays at Days 3 and 7. The level of cell mitochondrial activity of the four experimental groups was similar (Figure 4A). So, these results showed that the NMP treatment and/or the EMF exposure did not have an adverse effect on proliferation or mitochondrial activity (^†^ *p* > .05, ^††^ *p* > .05).

For examination of the membrane damage of Saos‐2 cells under different culture conditions, we performed LDH assays. The media was collected at Day 7 and analyzed. The NMP‐incorporated, EMF‐exposed, and NMP‐incorporated + EMF‐exposed groups did not have increased levels of LDH secretion (Figure 4B). No groups displayed a prominent difference in LDH (^†^ *p* > .05). Therefore, the LDH activity of the four experimental groups was similar, and it is believed that NMP and EMF did not induce cellular membrane damage.

### 3.4 Expression of osteogenic‐related genes

**Figure 5:**
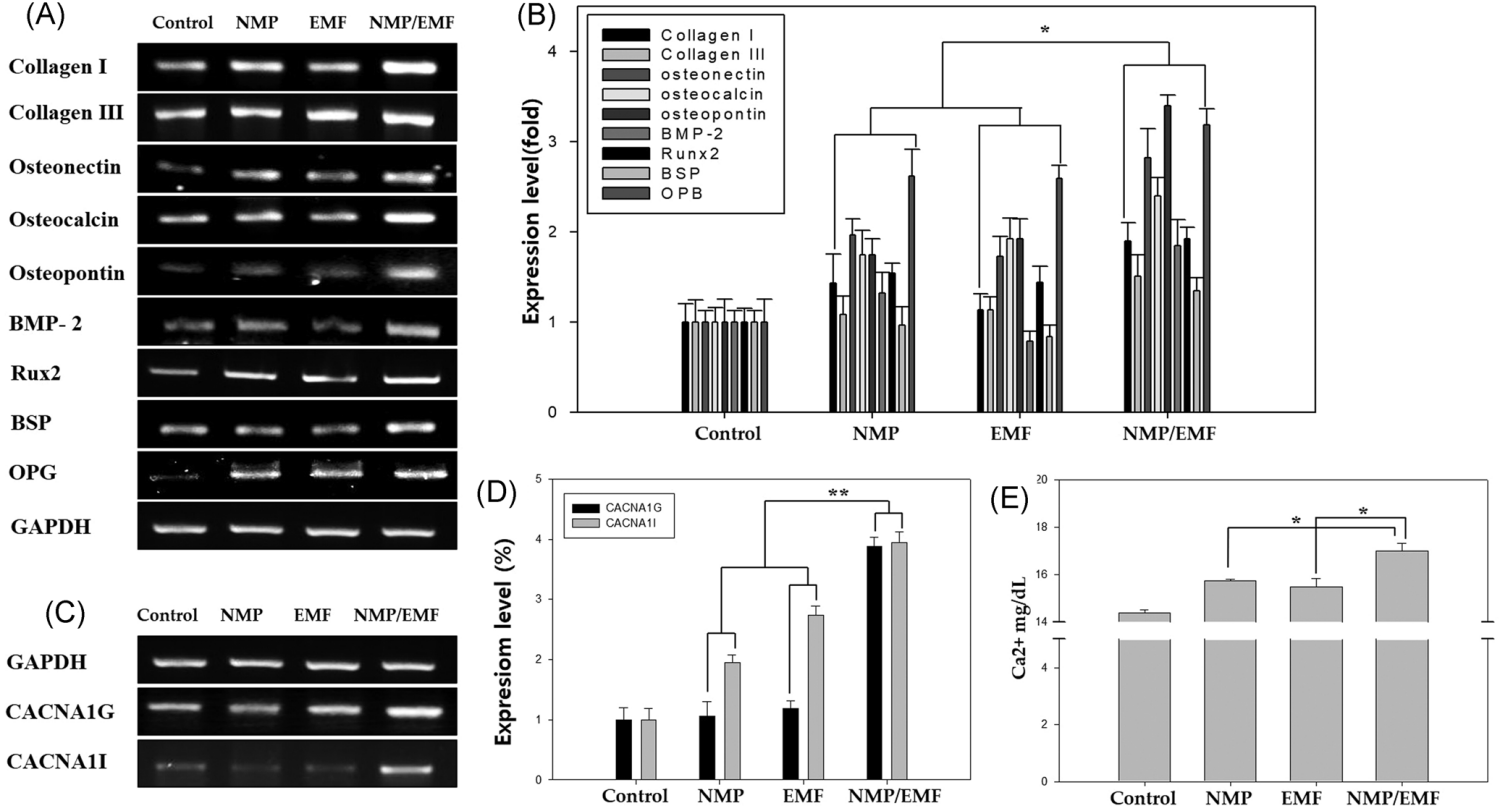
Level of gene expression detected by reverse‐transcription polymerase chain reaction (RT‐PCR) on Saos‐2 after culture for 7 days. (A) Electrophoretic RT‐PCR analysis of osteogenesis‐related genes and (B) quantitative analysis of messenger RNA (mRNA) levels of osteogenic markers in Saos‐2 cultures relative to GAPDH. (C) Electrophoretic RT‐PCR analysis of calcium channel‐related genes and (D) quantitative analysis of mRNA levels of osteogenic markers in Saos‐2 cultures relative to GAPDH. (E) The calcium concentration of Saos‐2 was detected by a Calcium Assay Kit. The calcium concentration of the nanomagnetic particle (NMP)‐incorporated + electromagnetic field (EMF)‐exposed group has increased about 10% compared with the control group (*p < .05, **p < 0.05)

For evaluation of the effect of NMP treatment and EMF exposure on Saos‐2 gene expression, total RNA was isolated from the cells of all groups, and RT‐PCR was carried out (Figure 5A). We examined the expression of each gene after normalizing it to that of GAPDH, and we reported the difference as the fold change (Figure 5B).

The major osteogenesis markers, collagen I, osteocalcin, and bone morphogenetic protein 2 (BMP‐2) were enhanced by more than 1.5‐fold, and osteopontin and osteoprotegerin (OPG) were highly expressed at the transcript level by more than threefold in the NMP‐incorporated + EMF‐exposed group compared with the control. The expression of the major bone matrix protein markers collagen I, collagen III, and osteocalcin were increased by 20%, and osteopontin, osteonectin. In the case of NMP‐incorporated + EMF‐exposed group, collagen I, osteocalcin, osteopontin, osteonectin, BMP‐2, OPG were highly expressed, and the expression levels of the bone matrix protein genes were significantly increased.

Also, the mRNA expression level of the transcription factor Runx‐2 was measured. Runx‐2 expression was enhanced in the NMP‐incorporated, EMF‐exposed, and NMP‐incorporated + EMF‐exposed groups by over twofold compared with the control groups. As shown in Figure 5B, the expression level of the osteogenic‐related genes was enhanced in the NMP‐incorporated + EMF‐exposed group compared with the other groups.

### 3.5 Evaluation of calcium channels

Calcium activation affects bone formation and osteogenic differentiation. Thus, calcium channel activation was measured by RT‐PCR. After 3 days of osteogenesis with NMP‐incorporation and/or EMF exposure, the mRNA levels of CACNA1G and CACNA1I were significantly increased (Figure 5C,D). This showed that NMP plus EMF exposure induced the activation of calcium channels.

To examine the mineralization of the Saos‐2 cells, a quantitative colorimetric calcium assay was performed on Day 7 (Figure 5E). The EMF‐exposed and NMP‐incorporated + EMF‐exposed groups exhibited higher calcium concentrations relative to the control group. Especially, the calcium concentration of the NMP‐incorporated + EMF‐exposed group increased by about 10% compared with the control group (*p < .05).

### 3.6 EMF and NMPs increase osteogenic protein expression in Saos‐2 cells

**Figure 6:**
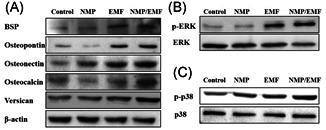
Western blot analysis of Saos‐2 after culture of 7 days. Whole cell lysates were immunoblotted with antibodies against (A) osteogenesis‐related proteins (bone sialoprotein, osteopontin, osteonectin, versican, osteocalcin), (B) ERK and p‐ERK, (C) p38 and p‐p38. EMF, electromagnetic field; NMP, nanomagnetic particle

We evaluated the expression of osteogenesis‐related proteins by Western blot analysis of Saos‐2 cells after culture for 7 days, using β‐actin as an internal control. The results shown in Figure 6A indicate that the expression of osteogenic proteins increased after NMP treatment and EMF exposure compared with the control group. In particular, in the NMP‐incorporated + EMF‐exposed group, all osteogenic proteins (including osteopontin, osteonectin, osteocalcin, and versican) were more abundantly expressed than in the control group.

To assess the mechanism involved in the osteogenesis of Saos‐2 cells, we evaluate the activation of p‐ERK and p‐p38 signaling. Western blot analysis revealed that the levels of phosphorylated ERK and phosphorylated p38 increased in the EMF‐exposed and NMP‐incorporated + EMF‐exposed group (Figure 6B,C).

### 3.7 Immunocytochemistry and immunofluorescence

**Figure 7:**
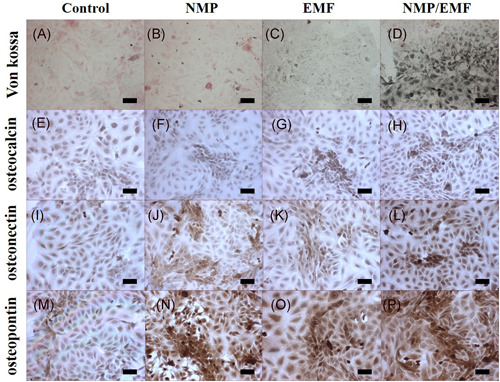
Immunocytochemical and Von Kossa staining of the Saos‐2 after culture for 7 days. (A–D) Von Kossa, (E–H) ostoecalcin, (I–L) osteonectin, (M–P) osteopontin; original magnification: ×100, scale bar = 100 μm. EMF, electromagnetic field; NMP, nanomagnetic particle [Color figure can be viewed at wileyonlinelibrary.com]

To further evaluate the protein expression of osteogenesis‐related proteins and mineralization, immunocytochemical staining was performed. We applied Von Kossa staining for the investigation of the mineralization of the Saos‐2 cells during osteogenesis (Figure 7A–D). The EMF‐exposed group exhibited a small amount of matrix mineralization, while the NMP‐incorporated + EMF‐exposed Saos‐2 cells exhibited stronger matrix mineralization compared with the other groups on Day 7. Osteocalcin was expressed in the NMP‐incorporated, EMF‐exposed, and NMP‐incorporated + EMF‐exposed groups compared with the control group (Figure 7E–H). The other osteogenesis markers (osteonectin, osteopontin) were more highly expressed in the NMP‐incorporated, EMF‐exposed, and NMP‐incorporated + EMF‐exposed groups compared with the control group (Figure 7I–P). The related staging intensity was scored as follows (Table 2): no or weak staining (−), low intensity (+), moderate intensity (++), and strong intensity (+++).

**Table 2:** Staining results of the osteogenic markers

	Control	MP	EMF	MP + EMF
Von Kossa	−	−	+	+++
Osteocalcin	−	+	+	++
Osteonectin	−	+	+	++
Osteopontin	+	+++	++	+++

Abbreviations: EMF, electromagnetic field; MP, magnetic particle.

**Figure 8:**
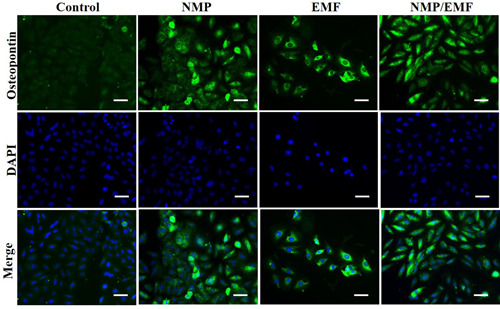
Immunofluorescence staining of Saos‐2 after culture for 7 days. Cells were fixed and labeled with anti‐osteopontin (green); original magnification: ×200, scale bar = 50 μm. EMF, electromagnetic field; NMP, nanomagnetic particle [Color figure can be viewed at wileyonlinelibrary.com]

Immunofluorescence staining of the Saos‐2 cells indicated the expression of the osteogenic‐related protein, osteopontin (Figure 8). Saos‐2 cells were fixed and labeled with anti‐osteopontin and DAPI (4′,6‐diamidino‐2‐phenylindole). Very weak signals were detected in the control group, while bright signals were observed in the NMP‐incorporated, EMF‐exposed, and NMP‐incorporated + EMF‐exposed group. Especially, many of the cells were strongly labeled in the NMP‐incorporated + EMF‐exposed group.

### 3.8 Micro‐CT 3D analysis

**Figure 9:**
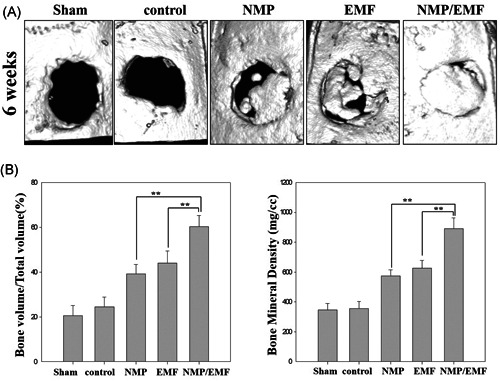
Micro‐computed tomography evaluation of bone regeneration in the rat calvarial defects at 6 weeks post‐experiment. (A) Top views of the reconstructed images; (B) bone volume/total volume in the defects; (C) bone mineral density in the defects. EMF, electromagnetic field; NMP, nanomagnetic particle. Mean ± *SD; n* = 3. *Significant difference between groups (*p < .05, **p < .01)

In the micro‐CT 3D images, new bone was observed at the margin of the defect in all groups. However, the bone density was higher in the NMP‐incorporated + EMF‐exposed group than in the NMP‐incorporated and EMF‐exposed groups at 6 weeks (Figure 9A). BV was significantly higher in the NMP‐incorporated + EMF‐exposed group than in the NMP‐incorporated and EMF‐exposed groups at 6 weeks (Figure 9B). The BV of the NMP‐incorporated + EMF‐exposed group at 6 weeks was 60.24 ± 4.9375%, which was higher than that of the NMP‐incorporated group (39.224 ± 3.94%) and the EMF‐exposed group (43.964 ± 5.50%) at 6 weeks. Also, the BMDs of the NMP‐incorporated + EMF‐exposed group were higher than in the NMP‐incorporated and EMF‐exposed groups at 6 weeks (Figure 9C).

## 4 DISCUSSION

The aim of this study was to investigate the osteogenic co‐effect of EMF and NMPs on Saos‐2 cells and a rat calvarial defects model. We evaluated the combination of these two parameters to induce synergic efficiency of osteogenesis in vitro and in vivo.

In this study, our data showed that no morphological changes of the Saos‐2 cells were observed, such as vacuole or signs of apoptosis, after EMF exposure (45 Hz, 1 mT, 8 h/day) and NMP treatment. Also, the LDH activity of the four experimental groups was similar. LDH is a cytoplasmic catalytic enzyme related to the reversible conversion of pyruvic acid to lactic acid, and it is released through the cell membrane when it is damaged.^28^

Many researchers have reported that radiofrequency exposure (RF, 900–1800 MHz) can induce mitochondrial DNA damage, reactive oxygen species (ROS), and apoptosis of cells. Other researchers have reported that exposure to more than 2 mT between 50 and 120 Hz or for a long time could induce an increase of mitochondrial damage or ROS.^29^ This is the result of cell damage and death caused by high frequency, high‐intensity magnetic fields, and long‐term exposure.

Some investigators reported that NPs can reduce cell viability and activity or did not have a positive effect on a dose, size, and surface character‐dependent manner.^30^, ^31^, ^32^, ^33^ Hou et al. showed that TiO_2_ NPs (size: 14–196 nm, dose: 0.05–0.2 mg/ml) decreased the migration and osteogenic differentiation of mesenchymal stromal cell (MSC),^32^ and Jiráková et al.^33^ reported that silica‐coated cobalt zinc ferrite NPs decreased cell proliferation of induced pluripotent stem cell cultures.

Our Fe_3_O_4_ NMPs were encapsulated with a PEG‐phospholipid shell to make them biocompatible, and the NMPs were inoculated at a concentration (50 µg/ml) that did not cause toxicity after cytotoxicity evaluation. Our results confirmed they were not cytotoxic to Saos‐2 cells and neither was the EMF exposure at 45 Hz, 1 mT, and 8 h/day during osteogenesis (Figure 4B).

Also, we examined the effects of EMF and NMPs on the expression of specific osteogenesis markers, such as BSP, osteocalcin, osteopontin, osteonectin, and osteoprotegerin. The gene expression and protein levels of these markers were all increased in the NMP‐incorporated + EMF‐exposed Saos‐2 cells (Figures 5 and 6). The osteogenesis markers of osteocalcin, osteopontin, osteonectin, and BSP were particularly highly expressed in the NMP‐incorporated + EMF‐exposed group. Osteocalcin and osteonectin are expressed in the earlier mineralization process, and osteopontin is an important marker of post‐mitotic osteoblasts.^34^, ^35^, ^36^ Also, BSP is expressed in osteoblasts^37^ and can function as a nucleator of mineralization in vitro and in vivo.^38^ In addition, the expression levels of major bone formation genes, collagen I, collagen III, Runx‐2, and bone morphogenetic protein 2 (BMP‐2), were increased in the NMP‐incorporated + EMF‐exposed groups. It is well known that BMP‐2 can induce the formation of bone^39^, ^40^ and it stimulates the expression of other osteogenic markers, such as osteopontin, osteocalcin, BSP, and alkaline phosphatase.^41^, ^42^ In our results, in the NMP‐incorporated + EMF‐exposed group, there was increased osteogenic‐related marker expression compared with the control and the other groups.

EMF effects have been previously investigated with osteoblasts and MSC. Martino et al. showed that EMF increased the ALP activity of Saos‐2 cells and mineral nodule formation at 0.9 mT, 15 Hz.^8^ Other studies have reported using EMF to induce the differentiation of stem cells into osteoblast‐like cells. Jazayeri et al.^16^ showed that EMF increased proliferation, Runx‐2 and OCN expression of rat MSC at 0.2 mT, 15 Hz. Kang et al.^43^ reported that EMFs at 30/45 Hz, 1 mT increased ALP, collagen I, and Runx‐2 expression when adipocyte stem cells were differentiated into osteoblasts. Furthermore, Lim et al. showed that exposure of alveolar bone‐derived MSC to various frequencies (10, 50, 100 Hz) of EMF (0.6 mT) strongly increased proliferation, mineralization, ALP, and calmodulin expression at 50 Hz.^22^

However, in every study, EMF did not increase the activity of osteoblasts. Chang et al. reported that low intensity (15 Hz, 0.1 mT) EMF decreased ALP activity and RANKL expression of mouse osteoblasts.^9^ Although the exact cause is unknown, the differences in these experimental results are expected to be due to the differences in the equipment, as all of the electromagnetic equipment applied were not off‐the‐shelf devices.

As well, research using NPs has been carried out in all areas of bio‐ and medical fields,^44^, ^45^ especially on the proliferation and differentiation of cells and wound healing using magnetic NPs.^30^ Choi et al. reported that 40 nm of chitosan‐conjugated gold NPs increased ALP, BSP, and OCN expression and enhanced the osteogenic differentiation of human adipose‐derived mesenchymal stem cells through Wnt/β‐catenin activation.^46^ Also, Zhang et al.^47^ showed that 45‐nm gold NPs induced ALP activity, mineralized nodule formation, and osteogenic‐related gene expression, but 5‐nm gold NPs reduced osteogenesis. Also, the effect of osteogenic differentiation using magnetic particles has been studied. Wang et al.^17^ reported that 30 nm of iron oxide NPs increased Runx‐2, BMP‐2, OMD, and ENG expression during induced osteogenic differentiation of human bone‐derived mesenchymal stem cells (hBMSCs).

Recently, the combined effects of magnetic NPs and magnetic fields have been studied for increasing cell proliferation and differentiation efficacy. Huang et al. manufactured hydroxyapatite scaffolds containing magnetic NPs and exposed the cells on them to EMFs to compare the osteogenic differentiation efficiency of MSC. In that study, there was no difference in cell proliferation or the expression of osteocalcin, only a slight increase in the expression of ALP and collagen.^23^ However, Paun et al. coated iron oxide NPs (20 nm) on a 3D scaffold and exposed cells on the scaffold to a magnetic field, then evaluated the osteogenesis of the MG‐63 osteoblast‐like cells. They reported that the amount of APL activity, osteocalcin, and Alizarin Red was increased in the magnetic field exposure group.^24^ Also, Russo et al. manufactured a PCL scaffold containing 20% of 25 nm of iron oxide (Fe_3_O_4_) and exposed cells on the scaffold to a magnetic field, and then analyzed the osteogenesis efficacy of MSC. Although there was no significant difference in cell proliferation, the expression of ALP and p‐ERK increased in cells exposed to the field but not NMPs compared with cells only exposed to NMPs. Also, the expression of ALP and p‐ERK increased strongly when the cells were exposed to both NMPs and EMFs. This can be interpreted to mean the osteogenic differentiation effect of EMFs is greater than that of magnetic NPs.^22^

The results of the above report are similar to our findings, and together they can be interpreted to have confirmed that a combination of magnetic NPs and magnetic fields has a synergistic effect on osteogenesis.

This result is also related to calcium channels and calcium concentrations. The mRNA expression of CACNA1G and CACNA1I in the NMP‐incorporated + EMF‐exposed group was higher than that of the other groups (Figure 5D). So, NMP‐incorporated + EMF‐exposed groups exhibited an increased calcium concentration. Many other investigators have reported that magnetic fields increase the intracellular Ca^2+^ concentration in lymphocytes, osteoblasts, neural stem cells, pituitary cells, and Jurkat cells.^36^ Also, 50 Hz of EMFs enhanced the expression of voltage‐gated calcium channels in the membrane of neuroendocrine cells.^48^, ^49^ Calcium is an important regulator of cellular activity, and enhanced calcium levels have important regulatory consequences in bone cells.^50^, ^51^ The calcium channels play fundamental roles in cellular responses to external stimuli in bone cells and activation of calcium channel signals is a key early feature of osteoblastic activation.^52^, ^53^ This means that the Saos‐2 cells' calcium channels were activated, indicating that osteogenesis and bone formation were upregulated.

NMP incorporation + EMF exposure induces signaling pathways through activation of p‐ERK, p‐p38, and Runx‐2. Several studies have mentioned that p‐ERK activation is an essential mediator of growth factor‐induced cell behavior and differentiation in various cell types, including osteoblasts.^54^, ^55^, ^56^, ^57^, ^58^, ^59^ The p38 signaling pathway plays an important role in the regulation of osteogenesis and osteogenic differentiation.^46^, ^60^ Also, Runx‐2 is involved in the production of bone matrix proteins, as it is able to promote the expression of major bone matrix protein genes, leading to an increase in immature osteoblasts differentiating from pluripotent stem cells; the immature osteoblasts then form immature bone.^61^, ^62^, ^63^ A related study reported that osteogenic differentiation of hBMSCs using 30 nm of iron oxide NPs was induced by the Runx‐2, ERK, and MAPK signaling pathways.^17^ In our study, we detected increased levels of phosphorylated ERK and phosphorylated p38 after 7 days of osteogenesis in the NMP‐incorporated + EMF‐exposed group.

We have confirmed the above results through immunocytochemical analysis. The NMP‐incorporated + EMF‐exposed group showed enhanced Von Kossa staining of Saos‐2 cells. Von Kossa staining is generally used to quantify mineralization.^64^, ^65^ Additionally, the results showed that exposure to EMFs and NMP treatments has the potential to facilitate osteogenesis based on the results of immunocytochemical staining and immunofluorescence (Figures 7 and 8). Saos‐2 cells more strongly expressed osteocalcin, osteopontin, and osteonectin in the NMP‐incorporated + EMF‐exposed group than the control group by immunocytochemical staining. In addition, in the immunofluorescence staining, we detected strong signals in the NMP‐incorporated, EMF‐exposed, and NMP‐incorporated + EMF‐exposed groups and weak signals in the control group. This means that NMPs and EMFs promote the mineralization of Saos‐2 cells.

To evaluate the bone regeneration efficacy of animals based on the in vitro results, the regeneration efficacy of NMPs and EMF were assessed using a rat calvarial defect model, and it was confirmed that there was a synergy effect of a combination of NMPs and EMF as shown in Figure 9.

Bone regeneration showed similar results to the in vitro analysis. We predicted that NMPs contained in a collagen scaffold would be phagocytosed by migrating osteoblasts or MSCs after transplantation, inducing cell activity or promoting their differentiation. Also, EMF exposure highly increased the osteogenic effect of NMP for bone regeneration. A previous study has reported that EMF exposure with MSCs containing NMP implanted in a spinal cord injury increased nerve regeneration compared with EMF exposure with only MSCs.

Taken together, these results suggest that treatment with NMPs or exposure to EMFs increases osteogenesis, and NMP incorporation in conjunction with EMFs exposure is more effective in enhancing osteogenesis. The results of this study demonstrated that NMPs can potentially be used on medical devices or scaffold materials, and that EMFs can be used for the rehabilitation of osteogenic wounds.

## 5 CONCLUSION

In this study, we have demonstrated that combined NMP treatment and EMF stimulation have a positive synergic effect on osteogenesis in vitro and in vivo. Also, NMP incorporation with EMF exposure was found to accelerate the expression of osteogenic markers and bone regeneration through calcium channel and p‐ERK activation. The results of our study demonstrated that these combined effects of EMFs and NMP have the potential to be used in clinical applications on bone disease and fractures.

## CONFLICT OF INTERESTS

The authors declare that there are no conflict of interests.
